# Competitive employer positioning through career path analysis: the case of the Swiss nursing sector

**DOI:** 10.1186/s12960-021-00586-z

**Published:** 2021-04-06

**Authors:** Remo Aeschbacher, Véronique Addor

**Affiliations:** 1 University of Fribourg (CH), Bd de Pérolles 90, 1700 Freiburg, Switzerland; 2 University of Applied Sciences and Arts Western Switzerland (HES-SO), Genève, Switzerland

**Keywords:** Comparative study, Employer positioning, Hospitals, Non-profit organisations, Nursing, Nurse, Home-care, Types of institutions, Turnover, Working conditions

## Abstract

**Background:**

The global shortage of nurses has caused strategic employer positioning and strengthened employer branding to become progressively relevant addressing the increased competition in the recruitment of nurses. This study provides competition-oriented strengths-and-weaknesses profiles for nurse attraction and attrition for the major types of healthcare institutions to advise on competitive employer positioning.

**Methods:**

We applied bivariate weighted logistic regressions with cluster-adjusted standard errors to evaluate 4844 employer changes of 3011 nurses participating in the *nurses at work* study, whereby the reasons to quit (RQs) acted as both predictors of the former and the follow-up type of employer. For each employer type, we introduce a coordination system allocating each workplace criterion along its push and implicit pull characteristics, given through the specific odds ratios, to derive different strategic implications for an organisation’s competitive nurse recruitment.

**Results:**

Depending on the employer type, workplace criteria were variously acting as push or pull factors in nurses’ career decisions.

**Conclusions:**

Nurses’ career choices are affected by experienced and presumed workplace characteristics associated with specific employer types. Becoming aware of these associations and experiences, employers should leverage workplace criteria with relatively strong pull or/and weak push characteristics by intensified communication measurements and criteria with relatively weak pull or/and strong push characteristics should be enhanced to a competitive level.

**Supplementary Information:**

The online version contains supplementary material available at 10.1186/s12960-021-00586-z.

## Background

Increased demand for care and problems in nursing supply, resulting from several factors including generational imbalances, technological advances, changes in the nursing job requirements and difficult working conditions (WCS), have led to a shortage of care professionals [1], which, in turn, facilitated voluntary turnover through ease of movement and compelled nurses to react more sensibly to poor WCS. Consequently, the competition to recruit nurses has increased worldwide, including in Switzerland [2–4]. Therefore, strategic positioning and strengthening employer branding is key.

However, data on which employer positioning in the health sector is based are limited to either specific comparative research on WCS aimed at hypothesis testing and therefore too narrow to apply on a holistic employer branding or firm-specific surveys and “best employer” studies, which are not generalisable.

### Study aim

Based on the theoretical and empirical groundwork of WCS across healthcare institutions, this study provides generic strengths-and-weaknesses profiles for major types of healthcare employers using a holistic inferential statistical approach.

Following secondary analyses of career statements originating from the *nurses at work* study [5], voluntary employer changes of nurses were assessed by first linking the various RQs to different types of left employers and, second, to use these RQs to predict the likelihood of different employer types being the follow-up employer. Thereof, we created the competitive strengths-and-weaknesses profiles for the largest types of nurses’ employers in terms of attraction (pull factors) and attrition (push factors) of nurses. Irrespective of queries on firm-specific branding and business strategy, practical strategic implications for each profile are derived.

Besides practical conclusions, the study advances the academic debate on institution-specific effects in the health sector by adding a causal perspective on the competing relationship between the left and subsequent employer in voluntary turnover. Furthermore, by theorising and measuring the effect of organisational context on turnover, we address a research gap in turnover research in the nursing literature [6].

### Theoretical perspective on workplace differences between healthcare employers

Studies that explain nurse turnover have applied conceptual models and theories that consider a wide range of variables involved at different stages leading up to voluntary turnover [6]. However, although studies in the field of general management have conceptualised voluntary turnover across the individual, workgroup and organisational analysis levels, researchers in the nursing field have mainly focused on studying turnover at the individual analysis level [6, 7]. Therefore, research has suggested integrating more theory from general management literature and other fields to build more integrated and powerful conceptual models studying nurse turnover [6, 7]. Thus, our conceptual and theoretical framework relates to various variables, discussing individual RQs depending on the type of employer that varies in size, activity field and ownership at the organisational and macroeconomic levels. In reference to an integrated conceptual turnover framework such as the Integrated Turnover Model proposed in previous research, the assessed RQs and organisational types are found at the first stage (related to the nature of the job) and the second stage (organisational context, person–environment context and job attitudes) [6, 7].

Our study aims at examining the variance in turnover reasons due to workplace differences across organisational types to advise on employer positioning in the labour market rather than explain voluntary turnover per se. Thus, we use theories that provide an explanatory foundation for the variance in turnover reasons, at an organisational and macroeconomic level.

Empirical studies of workplace differences across different healthcare employers often focus on one-dimensional comparisons such as for-profit versus non-profit hospitals or stationary versus ambulant practices and therefore lack a holistic theoretical framework to compare workplace and turnover variables across a wider spectrum of organisation types.

Supporting our aim to compare a greater range of institutions characterised across multiple dimensions, we consult a broader theoretical framework which respects the practical nature of the categorisation but examines underlying definitory variables.

Public hospitals, private for-profit hospitals, socio-medical institutions such as nursing homes (SOMEDs), home care services, private medical offices and general practitioners and non-profit organisations (NPOs) are the main types of employers for nurses in Switzerland. This categorisation is based on bundled variations in organisational size, activity type, and ownership and goal systems, which can therefore be regarded as mediators of institutional differences. Hence, a combined view on theories and concepts, as outlined in Table 1 [8], helps to comprehend potential differences in perceived workplace conditions across healthcare institutions.

**Table 1:** Mediators of perceived differences in workplace variables across institutions [8]

Underlying mediators of institutional differences	Description	Associated theories
Organisational size	Organisational size is a pivotal variable in classic organisational theory and considered a key mediator of differences in organisational structures, WCS and behaviour. According to the Formal Theory of Differentiation in Organisations and the Evolutionary Model of Organisation, size leads to the distinct characteristics of work, for example, by promoting functional specialisation, divided responsibility, wider control spans, standardisation, formalisation and less centralisation [9–11]. More recent economic theories have described the effects of organisational size on WCS, particularly the positive effect on compensation, training, promotion opportunities, job security and the negative effect on participation, meaningful work, worker’s confidence, autonomy and job satisfaction [12–21]	e.g. Formal Theory of Differentiation in Organisations [9]; Evolutionary Model of Organisation [10]; High-performance work systems [13]
Activity type	Activity type refers to the type of treatment, patients and locations a healthcare provider is associated with, which, according to the self-determination theory and the job characteristics theory, can impact work motivation, exhaustion and overall job satisfaction by offering various levels of personal-identity-fit, perceived impact on others, meaning and interestingness, as well as autonomy and feedback [24–24]. Moreover, with regard to the effect of patient types, social interaction theories suggest that the quality of nurse–patient relationships affects nurses’ well-being and work strain by positive and negative regulation of emotions [25, 26]. Finally, context variables indirectly impact WCS by being linked to activity type. For example, in Switzerland, different billing systems for various treatments affect nurses’ WCS by promoting cost-savings [27, 28]	e.g. Self-determination theory [22, 25]; Economisation at hospitals [27, 28]; Job characteristics theory [23],
Ownership and goal system	Ownership and goal systems refer to institutions being in either private or public ownership and following for-profit or non-profit objectives. As for-profit, non-profit and public organisations typically act consistent with different macroeconomic roles [29], they promote different workplace characteristics and therefore offer different intrinsic and extrinsic stimuli for workers’ motivation. From the self-determination theory perspective, promoting autonomy, relatedness and competence increases workers’ intrinsic motivation [22] and job satisfaction [30, 31]. NPOs offer more autonomy because of the relative absence of competitive or legislative/regulatory pressure, compared with for-profit or public organisations, while both public organisations and NPOs can offer more relatedness at work than their for-profit counterparts because of public service motivation [32, 33]	e.g. Three-Sector Economy [27], Public Service Motivation [33], Self-determination theory [22]

### Nurses’ WCS and turnover across different types of organisations

Previous comparative research of WCS across various institutions can be presented well following the structure of the theoretical discussion. Studies comparing organisations of different sizes show that working with larger healthcare employers, such as hospitals, is associated with more work strain and burnout, less autonomy and participation, and more regulation and burnout [34–38].

Comparison studies of work settings across various activity types found that nurses working with challenging patients, typically in long-term care (i.e. SOMEDs), experience more stress and burnout [39, 40], but show more organisational commitment and higher professional identification [36]. Nurses working in outpatient or home care (i.e. home care services) in contrast to stationary care experience more autonomy, less regulation [36, 41], less work strain [39] and job satisfaction [36] and report higher meaning of work [42].

Research comparing workplaces with various types of ownership and goal systems found that nurses in the private sector, rather than the public sector, experience less administrative workload [27], less violence, more recognition [35], higher commitment [43] and greater job satisfaction [44]. However, nurses at public hospitals are more likely to be satisfied with salary and experience more job stability and employee benefits [45]. Nurses working at NPOs report less burnout [46] while earning more [47] and reporting greater well-being [48]. Generally, employees at private medical offices experience more autonomy and job satisfaction [32, 49].

Finally, using the introduced typology of the main six employers of Swiss nurses—public hospitals, private hospitals, private medical offices, socio-medical institutions, non-profit organisations and home care services—a recent comparative study shows that nurses working in private hospitals and public hospitals were less likely to experience autonomy and worktime flexibility than those working at smaller healthcare employers, whereas work at public hospitals, rather than at private hospitals, was associated with more stress, yet associated with more satisfaction with salary and advancement opportunities. Compared with other workplaces, SOMEDs were associated with more alienating WCS and lower job satisfaction, but with more participation and decision-making. In contrast, private medical offices were associated with a milder work environment and more social support than other institutions. Finally, working at NPOs and home care services was associated with higher degrees of autonomy, recognition, organisational commitment and job satisfaction [8].

Besides the assessment of WCS, nurse staffing and turnover were the focus of several studies, especially because they are linked to patient safety, health outcomes and mortality [52–54]. With lower overall job satisfaction, the tendency for turnover is greater [57–58]. More specifically, strong positive predictors of turnover or turnover intent in nursing are job stress, workload, emotional exhaustion and burnout [59–66]. Further factors significantly linked to turnover are satisfaction with salary, career opportunity and professional development [64, 65, 67], organisational commitment [58, 63, 68], team culture, peer networks and leadership [59, 63, 65, 67, 69–72], job characteristics such as non-nursing tasks [66], meaningful work [57], job complexity [63], perceived patient safety, sex (i.e. being male) [66], autonomy, participation and recognition [57, 63, 64].

Research on nurses’ turnover by type of institution is scarce compared to studies of turnover reasons in general. However, even if not differentiating between different RQs, several studies compare turnover intention or actual turnover across different employer types and settings. Nursing staff in nursing homes and hospitals is found to consider leaving nursing more often than those in home care [36], staff in hospitals more often considers leaving nursing than in primary outpatient care and nursing homes [73] and nursing staff in private hospitals considers leaving nursing to a higher degree than those working in psychiatric hospitals [74]. Moreover, nurses in nursing homes report greater intention to leave than nurses working in private or public hospitals [75]. Nurses in geriatric care are more likely to quit their job [76]. Finally, smaller work units, outpatient units and day care settings are associated with lower staff turnover [72]. Thereby, nurses working in home care may have a lower turnover propensity because workers in low-wage home care perceive more meaning and dignity. After all, it is supposed that nurses enter home care after quitting an alienating job, within or outside the healthcare industry [42].

## Methods

### Data

We used data from the *nurses at work* study [5], a retrospective longitudinal cohort study of career paths of nurses who worked in Switzerland between 1970 and 2014. 15,301 nurses answered the online questionnaire between September 2014 and February 2015. The survey included questions about experienced WCS and RQs as well as it included a parallel section on simultaneous personal events, socio-demographic data and personality type.

### Sample

To evaluate the pull and push characteristics of workplace criteria, we focused on voluntary staff turnover between the six main types of healthcare employers who employ roughly 90 per cent of all professional nurses, namely public hospitals, private hospitals, private medical offices, SOMEDs, NPOs and home care services [8]. After excluding movements within the same type of institution and movements from workplaces at which individuals worked less than one full day and/or had a tenure below one month, we analysed 4844 movements from 3011 nurses [see Additional file 1 for more details].

### Reasons to quit

In the *nurses at work* study, respondents were asked to state why they left each of their former employers. The question ‘For what reasons did you leave this job?’ was only prompted if the turnover was voluntary, e.g. due to dissatisfaction with the job, due to a job offer or due to personal reasons. With the instruction ‘Please state any reasons that led you to leave the position’, the respondents were asked to select multiple RQs from a catalogue of 28 statements which contained items concerning the WCS as well as some items addressing reasons coming from outside of the organisation (e.g. job offer). Respondents could answer with *yes* or *no* or *does not concern me* in the case of a workplace could not be assessed in terms of this criterion. The catalogue of RQs concerning characteristics of the workplace or the tasks were, mostly, directly derived from validated variables addressing WCS [5].

### Statistical analysis

#### Principal component analysis

To reduce the number of RQs and ease results’ visualisation, we applied principal component analysis (PCA) based on a tetrachoric correlation matrix and obtained orthogonal (independent) factors via varimax rotation. The Kaiser–Meyer–Olkin measure of sampling adequacy indicated that PCA was appropriate. Thus, in the final analysis, we assessed overall 18 RQs (see Table 2).

**Table 2:** Measures of reasons to quit

Latent construct name	Item name (binary scale: 0 = no; 1 = yes)	Statement
Non-nursing tasks avoidance	Non-nursing tasks avoidance (RQ1)	I had to do too many non-nursing tasks (for example, picking up the food plateaus)
Work hours	Work hours (RQ2)	My working hours were too inconvenient
Care quality	Care quality (RQ3)	I had the impression that the quality of the care and/or the patient safety were insufficient
Exhaustion (KR-20 = .70)	Stress (RQ4)	The work was too stressful, too much physical and/or mental stress
	Professional exhaustion (RQ24)	I was in a state of professional exhaustion
	Health problems (RQ25)	I had a health problem
Violence	Violence (RQ5)	There was increased verbal or physical violence from patients/relatives to nurses
Wish for change (KR-20 = .50)	Interest in other profession (RQ6)	I was interested in another profession
	Professional development wish (RQ7)	I wanted to professionally develop
	Education wish (RQ27)	I wanted to do an education / a training
Skill-use opportunity	Skill-use opportunity (RQ8)	I was not able to use my nursing education and specific skills
Self-actualisation (KR-20 = .67)	Autonomy (RQ9)	I could hardly work independently
	Participation (RQ10)	I had very little opportunity to decide (about the patient care, the department, the company)
Mobbing	Mobbing (RQ11)	I was bullied at the workplace
Interesting job offer	Interesting job offer (RQ12)	I got an interesting job offer
Advancement (KR-20 = .64)	Training opportunities (RQ13)	I could hardly benefit from further training opportunities
	Career opportunities (RQ14)	I did not have enough career opportunities
Team (KR-20 = .79)	Team mood (RQ15)	The team mood was bad
	Team cooperation (RQ16)	The cooperation in the team was unsatisfying
Superiors (KR-20 = .78)	Superiors' support (RQ17)	I did not get enough support from my superiors
	Recognition (RQ18)	My work has not been sufficiently recognised
Organisational commitment	Organisational commitment (RQ19)	At that time, I had no sense of belonging to this organisation
Professional identification	Professional identification (RQ20)	At that time, I was only slightly identified with the nursing profession
Work–life balance (KR-20 = .67)	Work–life balance (RQ21)	I wanted more time for my private life (for example family, travel …)
	Taking care of children (RQ22)	I wanted to look after my children
Moving to a new house	Moving to a new house (RQ23)	I moved houses
Salary	Salary (RQ26)	The compensation and/or social benefits were unsatisfying

#### Regression analysis

Research on turnover at the organisational level and addressing staff migration on macroeconomic level states push-and-pull factors. Push factors refer to the factors leading to dissatisfaction at the workplace, which in turn leads to turnover. In contrast, the actual situation on the labour market or the available alternatives has been considered pull factors [77]. However, this study argues that a criterion leading to turnover from a specific employer can be considered an implicit pull factor from the follow-up employer’s perspective [78]. Although this indirect measure of pull factors can be considered a limitation—as the reasons to join employers were not surveyed—the approach benefits the interpretation of results. Implicit measurement of pull factors ensures the conceptual integrity when comparing the push-and-pull factors and informs about the competitive significance of the factor for a specific organisation type as it earlier led to turnover from another. With a direct assessment of pull factors associated with a subsequent employer, respondents would omit factors, especially unsatisfied hygiene factors associated with the former employer, that led to the employer change, in the first place.

Since we only looked at voluntary turnovers, the underlying axiom is that the follow-up employer is expected to perform better in the criterion that led to the withdrawal from the previous employer [79]. Thus, a criterion, on the one hand, can be either a significantly more frequent RQ (strong push argument) for nurses of a certain type of organisations, a significantly less frequent RQ (weak push argument), or an average frequent RQ (average push argument), compared to turnovers from other types of organisations. On the other hand, the criterion can in the same way be characterised as strong, weak, or average pull argument by estimating the likelihood of a certain type of employer being chosen after leaving the former employer because of this very criterion.

**Fig. 1:**
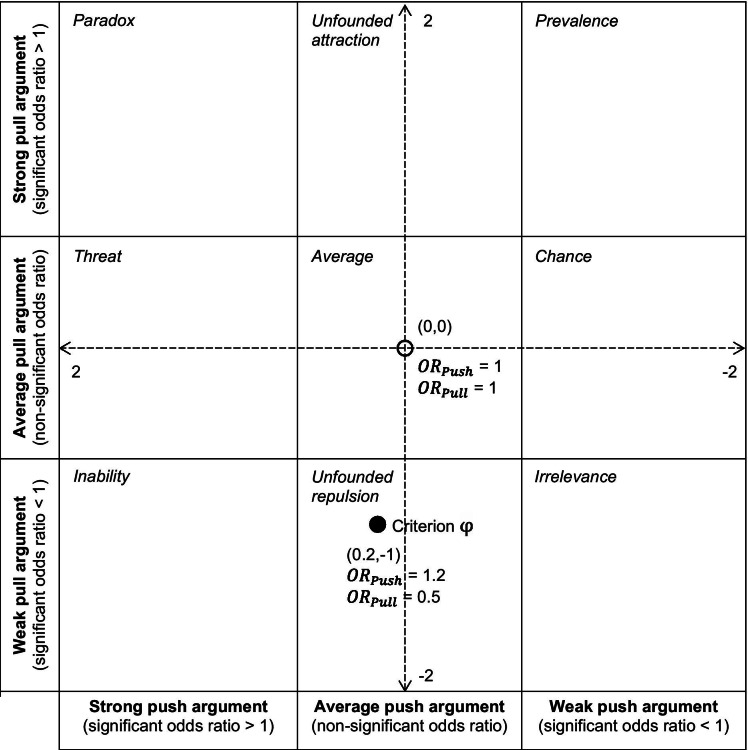
Odds ratios (OR) allocating each criterion to one of nine strategic fields

Figure 1 illustrates a coordination system along which push-and-pull characteristics of a criterion can be directly displayed allocating the specific criterion to one of nine strategic fields. The fields are named after what specific factors in these fields should be perceived as by managers. For each field, we suggest different generic employer reactions, which can be retrieved in detail from Additional file 2.

In our analysis, the location of the criteria for each type of organisation relied on the results of 2 $\times$ 18 bivariate weighted logistic regressions (with cluster-adjusted standard errors), whereby the specific RQ first acted as predictor of the organisational type being the prior employer (defining the push characteristics), and second, acted as predictor of the specific organisational type being the follow-up employer (defining the implicit pull characteristics). In each of the two models, the specific type of organisation (as prior and follow-up employer, respectively) was coded as dependent dummy variable, 1 meaning that the specific type was left (push model) or chosen (pull model), 0 meaning that another type was left (push model) or chosen (pull model). Since, in the reference group (0), more frequently involved types of organisations also would have shaped the characteristics of the reference group more heavily, the cases were weighted so that each type of organisation affected the reference group of the dependent variable in an equal manner.

We employed odds ratios ($ORs$) indicating the degree to which the odds of leaving (${ORs}_{\mathrm{Push}}$) or going to (${ORs}_{\mathrm{Pull}}$) a specific type of organisation is increased ($OR>1$) or decreased ($OR < 1$) when a nurse indicated a certain RQ. Thus, when regressing a certain type of former employer on a specific RQ was associated with a significant $OR$ above 1, the RQ was considered as a strong push factor, relatively to its peculiarity with the turnovers from other types of former employers. When the RQ was associated with a significant $OR$ below 1, it was considered as a weak push factor, relatively to its peculiarity with other types of former employers. The same principle applies to the type of follow-up employer we regressed on the RQ associated with the former type of employer, in this respect acting as pull factors. If there was no significant relative prevalence or infrequency of a turnover argument associated with the specific type of former or follow-up employer, it was considered as an average push or pull argument, respectively.

The exact coordinates of the criteria in the diagram indicating competitive strengths-and-weaknesses were determined by the odds ratios ($OR$) as multipliers of the odds if they were equal to or greater than 1, or dividers $\left(\frac{1 }{OR}\right)$ of the odds in the case $OR$ were below 1. Starting from a neutral point in the centre of the diagram (0,0), multiplier characteristics were displayed as $OR-1$, in the direction of the *strong* predicate, and dividers were displayed as $\left(\frac{-1 }{OR}+1\right)$, in the direction of the *weak* predicate.

Thus, for a for criterion *φ* the *x* coordinate was defined as follows:

$$\left( {OR_{{{\text{Push}}}} \ge 1 \to x_{{{\text{Criterion}} \varphi }} = OR_{{{\text{Push}}}} {-}1} \right) \wedge \left( {OR_{{{\text{Push}}}} < 1 \to x_{{{\text{Criterion}} \varphi }} = \frac{ - 1 }{{OR_{{{\text{Push}}}} }} + 1} \right)$$

And the *y* coordinate for criterion *φ* was defined as follows:

$$\left( {OR_{{{\text{Pull}}}} \ge 1 \to y_{{{\text{Criterion}} \varphi }} = OR_{{{\text{Pull}}}} {-}1} \right) \wedge \left( {OR_{{{\text{Pull}}}} < 1 \to y_{{{\text{Criterion}} \varphi }} = \frac{ - 1 }{{OR_{{{\text{Pull}}}} }} + 1} \right)$$

## Results

**Fig. 2:**
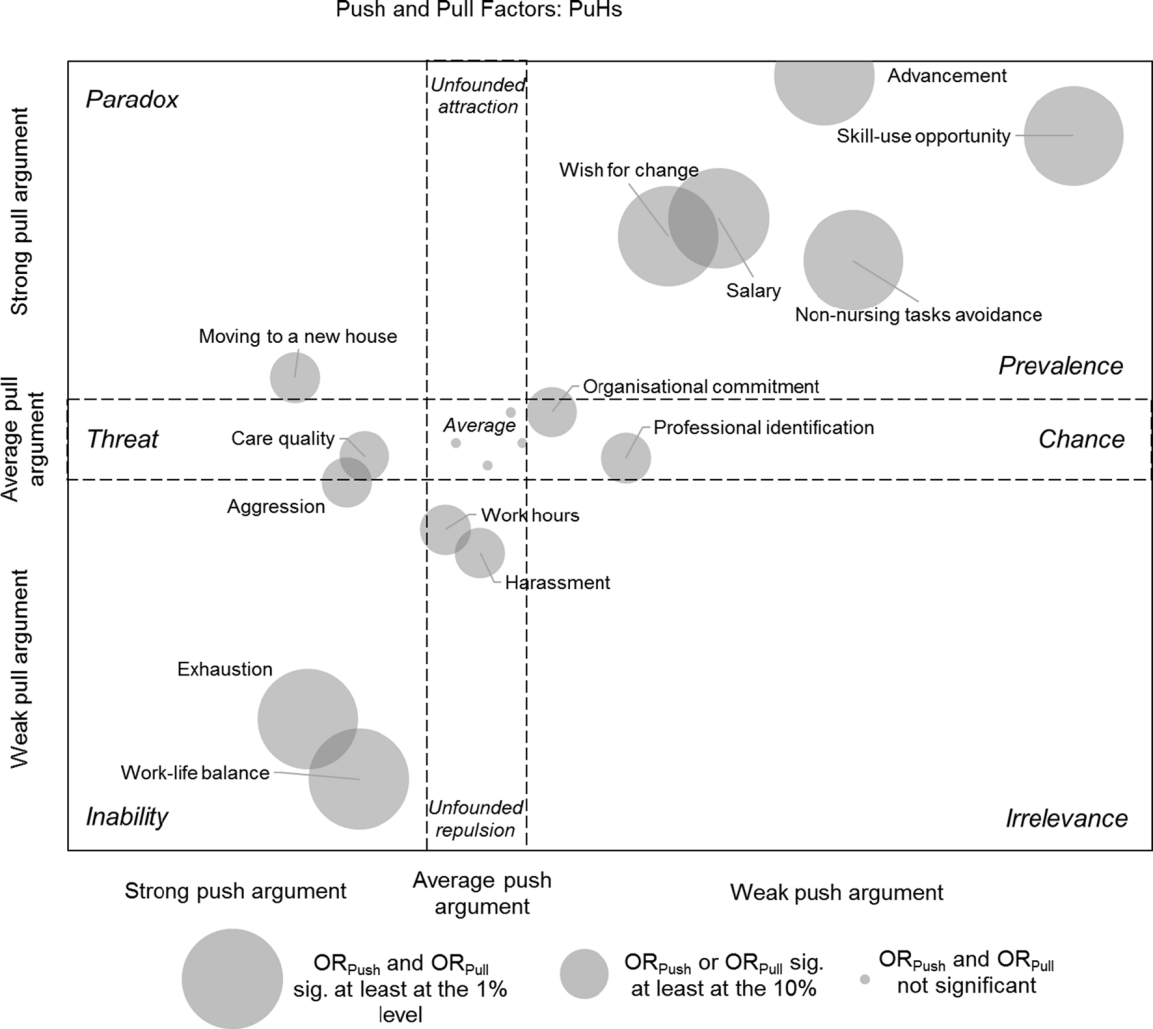
Push-and-pull factors associated with working at public hospitals. *Notes*: coordinates based on transformed odds ratios of cluster-robust weighted bivariate logistic regressions determining the push-and-pull characteristics of each criterion

Figure 2 shows the specific competitive strengths-and-weaknesses diagram for public hospitals, while Additional file 3 presents the diagrams for the other employer types. Table 3 holds the aggregated results.

**Table 3:** Summary of competitive turnover profiles

	Employer strengths			Employer weaknesses			Paradox (strong pull argument; strong push argument)	Irrelevance (weak pull argument; weak push argument)
	Prevalence (strong pull argument; weak push argument)	Unfounded attraction (strong pull argument; average push argument)	Chance (average pull argument; weak push argument)	Inability (strong push argument; weak pull argument)	Threat (strong push argument; average pull argument)	Unfounded repulsion (average push argument; weak pull argument)		
Public hospitals	Advancement Skill-use opportunity Wish for change Pay Non-nursing tasks avoidance		Organisational commitment Professional identification	Exhaustion Work–life balance	Care quality Aggression	Work hours Harassment	Moving to a new house	
Private hospitals	Aggression Non-nursing tasks avoidance	Pay	Exhaustion Work hours Skill-use opportunity			Self-actualisation		Professional identification Work–life balance
Private medical offices	Work hours	Work–life balance	Exhaustion Care quality	Wish for change	Skill-use opportunity Advancement Pay Self-actualisation Non-nursing tasks avoidance Professional identification			Aggression Moving to a new house
Socio-medical institutions (SOMEDs)		Harassment Moving to a new house		Interesting job offer Advancement	Team Self-actualisation Work hours Skill-use opportunity Non-nursing tasks avoidance Organisational commitment	Wish for change Pay	Exhaustion Aggression Care quality Superiors	
Non-profit organisations (NPOs)	Wish for change	Harassment	Interesting job offer Self-actualisation			Work hours Skill-use opportunities		Advancement Pay Care quality
Home-care services	Self-actualisation Care quality	Exhaustion Team	Wish for change				Work hours	

## Discussion

### Public hospitals

Figure 2 indicates that nurses were more likely to work for a public hospital as a follow-up employer (strong pull arguments) when they quit their jobs because of low *skill-use* and *advancement* opportunities, when they wanted a *professional change*, when they were dissatisfied with *salary* and/or when they quit because of too many *non-nursing tasks*. In turn, these reasons were, as well, less indicated as RQ by nurses who left public hospitals (weak push arguments) and can therefore be considered type-specific strengths.

However, there are several deficits in WCS that were more frequently indicated as RQ and were less likely to act as arguments attracting new employees (*Inability*). For example, nurses who quit because of *exhaustion* and/or a poor *work–life balance* were more likely to have worked at a public hospital.

### Private hospitals

Nurses who left the former organisation because of low *salary*, too much *non-nursing tasks*, *aggression* and/or bad *care quality* were more likely to go to a private hospital. However, since *care quality* was also measured a strong push argument (*Paradox*), one may argue that private hospitals might be very heterogeneous regarding this aspect. Furthermore, nurses at private hospitals were more likely to come and go as a consequence of changing the place of residence.

What seems favourable to private hospitals is—moreover—that nurses were less likely to quit because of *exhaustion*, inconvenient *work hours* and low *skill-use opportunity* (*Chance*). Furthermore, *work–life balance* and *professional identification* were measured weak push arguments. But since these were also less likely to pull nurses towards private hospitals (*Irrelevance*), it may suggest that nurses at private hospitals are not overly seeking increased *professional identification* or enhanced *work–life balance*, in the first place.

### Private medical offices

Nurses working at private medical offices were more likely to leave because they could not use their *skills*, had low *advancement opportunities* and did tasks that they considered to be *non-nursing activities*. They were also more likely to leave because of low *self-actualisation* and dissatisfaction with *salary* as well as lowered *professional identification*. Yet, these criteria were no weak pull arguments (*Threat*), what may indicate hidden disadvantages or heterogeneity within the employer type. What can be considered—however—as important competitive weakness (*Inability*) concerns the inability to adequately satisfy nurses’ *wish for change*.

However, nurses at private medical offices were less likely to leave because of *exhaustion* and *care quality*, which can be considered as generally important reasons to leave a healthcare institution (see Additional file 4). Nevertheless, it could not be significantly measured that these also acted as strong pull factors (*Chance*). However, considered as a competitive strength—working as a strong pull argument while being a weak push argument (*Prevalence*)—are *work hours*. Finally, *work–life balance* is considered a strong pull argument, as well. That *moving to a new house* was located in the field *Irrelevance* field implies that nurses at private medical offices are either willing to accept longer distances between their home and their workplace or that they change their place of residence less frequently.

### SOMEDs

In comparison to other nurses, it was more likely for SOMED employees to leave the organisations because of harsh WCS captured in several criteria. This was also reflected in the competitive disadvantage (*Inability*) that SOMEDs seemed to lack on providing *interesting job offers* and hence were losing nurses to more appealing workplaces. Moreover, that *moving to a new house* was measured a strong pull factors suggests that nurses are more likely to be urged to work for SOMEDs.

Looking at further strong pull factors of SOMED workplaces, we paradoxically observed that the factors like *exhaustion*, *care quality* and *superiors*—while strongly pushing nurses away from SOMEDs—also were more likely to attract nurses (*Paradox*). This may either suggest that there is a sub-segment of SOMED workplaces that offers good conditions regarding these aspects or that nurses have false expectations towards theses specific WCS at SOMEDs.

### NPOs

NPO workplaces were more likely to attract nurses with a *wish for change* of work content while still working as nurses (*Prevalence*). Additionally, NPOs seem to prevail in providing *interesting jobs*, with much autonomy and participation (e.g. *self-actualisation*), although the criteria were yet located in the field of *Chance*. This implies that human resource management (HRM) at NPOs should stress these aspects more in their communication measurements to make them strong pull arguments. That personality and distinct motivational patterns could play a crucial role in attracting NPO nurses is supported by the irrelevance of factors that were of central interest in other employer profiles: *salary*, *advancement* and *care quality*.

### Home-care services

In comparison to other nurses, nurses in home care services were not more likely to quit because of a specific reason, except because of inconvenient *work hours*. The fact that it was at the same time measured a strong pull factor (*Paradox*) is explainable by the ambivalent nature of the criterion (need variance). Like private medical offices, home care services offer shifts that clearly differ from hospitals (e.g. less night shifts needed).

Moreover, nurses who quit their former employers because of *exhaustion*, *team*-related aspects, *care quality* and lack of *self-actualisation* were more likely to choose home care services as their follow-up employers. The latter two criteria were, in addition, also measured weak push arguments making them strong competitive advantages (*Prevalence*) which seems even more favourable since these factors are generally rather common RQs (see Additional file 4).

## Conclusion

### Practical implications

In the rising competition for well-trained employees, nurses choose their follow-up employer deliberately according to criteria they ascribe to specific types of organisations. The turnover profiles presented in this article not only show that there is need for competitive agility but reveal, as well, where different types of employer should begin to improve and contrast, independently from their firm-specific branding or their business strategies. Depending on the position of workplace criteria in the specific competition-oriented diagram, employers may use the generic strategic HRM and employer branding recommendations suggested in Additional file 2. Criteria located in the *Prevalence* field are strong competitive advantages and can be communicated highly credibly to attract nursing workforce. Opposed to these, criteria in the *Inability* field are type-innate weaknesses which should be supervised carefully. Although HRM may never turn them into competitive advantages, these weaknesses should be enhanced to an acceptable level to reduce turnover. Criteria located in the *Paradox* field need further analysis since they underlie effects of either high workforce sensitivity, workforce heterogeneity or distorted employer perceptions on the labour market. Criteria allocated in the *Irrelevance* field are comparatively less involved in both nurse turnover and attraction. Hence, in competitive comparison, these criteria do not ask for immediate treatment by the HRM. However, criteria which reach significance either in their competitive push or pull dimensions but not on both indicate either positive or negative branding potential in one dimension, which should be addressed appropriately.

As the results bring more transparency to the nurses’ labour market and present the push-and-pull factors of major groups of health employers, this study may interest human resource management and employer branding professionals, support nurses in their career decisions and guide political and economic representatives of specific types of employers or nurses in Switzerland (i.e. professional associations and industry organisations) in their discussion of nurses’ WCS.

Although healthcare systems and labour markets differ across countries, our findings may be relevant for future research and HR managers abroad. After all, the discussed theoretical effects of institutional characteristics such as size, activity field and goal system are less bound to the political and socio-economic context. Moreover, the basic typology of major nurse employers may be found worldwide (hospitals, doctor’s offices, home care services and for-profit vs. non-profit offices). Furthermore, although correlations for specific variables involved in turnover decisions vary across countries and therefore hint at the importance of national contexts [80], similar outcomes and unidirectional effects for key predictors of turnover intention, such as job satisfaction, stress or burnout, are shown in literature reviews and cross-country studies of nurse turnover and working conditions such as RN4CAST or the NEXT study [52, 61, 80–82].

### Limitations and implications for future research

Our research has some limitations. The surveyed sample may suffer self-selection bias and therefore lack representativeness because a sampling frame such as a registry of professional nurses was not available. Furthermore, while RQs were surveyed with each left employer, the questionnaire did not address the reasons why a certain employer was joined. This limitation was overcome using implicit pull factors as described earlier. Moreover, although the survey considered several RQs, the literature suggests further unassessed reasons for a voluntary turnover that could account for additional variance in career decisions. For example, nurses may be pulled out of the organisation by former peers and join them to another [6, 83].

We introduced a method to map results based on inferential statistical analyses, which can be adapted to any competitive field and extended with any control variables. Furthermore, the approach offers high automation potential for career platforms such as LinkedIn, which have already implemented analytics for organisations to identify recruitment competitors based on employee turnover. Surveying push-and-pull factors may result in the automated creation of competitive employer profiles online.

Future research may examine employer changes qualitatively and evaluate individuals’ expectations and experiences before and after the change. Moreover, as this study is limited to previous and follow-up employers only, future studies may explore the career paths of nurses across various healthcare employers holistically to shape employer branding with a better understanding of what nurses seek in their long-term careers.

The analysis revealed imperfect transparency in the applicants’ market regarding WCS with specific employers, which can be both useful and unfortunate for HRM. Moreover, the results indicate that different personality types attracted by specific employer types relativises the importance of certain WCS and their involvement in career decisions, which needs further research.

The nurse data were collected as early as 2014; hence, timely representativeness may be limited. However, because of the large sample size and data covering careers ranging back decades, our findings may rely on more structural and innate characteristics in the health sector and labour market while having relativised short-term changes and extraordinary events to insignificancy. Underlying theories have been proven valid over time and career decisions are affected by long-term experiences [84]. Finally, studies and literature reviews from different decades have shown the persistence of the main challenges and key factors associated with nurse staffing, turnover and working conditions, such as stress, burnout and organisational commitment as key predictors of turnover intention [81, 85, 86].

Finally, the competitive positioning of nurses’ employers in other countries may be assessed in future studies, providing more information about the robustness of the competitive push-and-pull profiles of employers across different healthcare systems, political and economic environments, hospital and professional standards and work regulations.

## Supplementary Information

**Additional file 1:** Movement sample composition

**Additional file 2:** Profiling nurse turnover: interpreting criteria allocation and deriving strategic reactions

**Additional file 3:** Strengths-and-weaknesses diagrams for private hospitals, private medical offices, SOMEDs, NPOs, and home care services

**Additional file 4:** Relevance of reasons to quit involved in cross-typical turnover

## Data Availability

The nurses at work database was made available by the authors upon submission of a research protocol.

## Abbreviations

AF: Additional file
HRM: Human resource management
NPOs: Non-profit organisations
PCA: Principal component analysis
RQ(s): Reason(s) to quit
SOMEDs: Socio-medical institutions
WCS: Working conditions
