# Supplementary material for: Competitive employer positioning through career path analysis: the case of the Swiss nursing sector
Source: Hum Resour Health. 2021 Apr 6;19:47. doi: 10.1186/s12960-021-00586-z (PMC8025559; doi:10.1186/s12960-021-00586-z)
Supplement: Supplementary file 1 — Additional file 1: Movement sample composition [file 12960_2021_586_MOESM1_ESM.docx]

***Additional File 1: Movement sample composition***

|  | *Former employers* | | *Follow-up employers* | |
| --- | --- | --- | --- | --- |
|  | *N* | *%* | *N* | *%* |
| Public hospitals | 2,302 | 48% | 1,469 | 30% |
| Private hospitals | 880 | 18% | 835 | 17% |
| Private medical offices | 142 | 3% | 133 | 3% |
| SOMEDs | 815 | 17% | 1137 | 23% |
| NPOs | 198 | 4% | 221 | 5% |
| Home care services | 507 | 10% | 1,049 | 22% |
| Total movements from 3,011 individuals | 4,844 | 100% | 4,844 | 100% |
