# Supplementary material for: Competitive employer positioning through career path analysis: the case of the Swiss nursing sector
Source: Hum Resour Health. 2021 Apr 6;19:47. doi: 10.1186/s12960-021-00586-z (PMC8025559; doi:10.1186/s12960-021-00586-z)
Supplement: Supplementary file 2 — Additional file 2: Profiling nurse turnover: interpreting criteria allocation and deriving strategic reactions [file 12960_2021_586_MOESM2_ESM.docx]

## Additional File 2: Profiling nurse turnover: interpreting criteria allocation and deriving strategic reactions

The following figure displays the nine fields to which workplace criteria potentially is allocated according to the method described in the Methods section. Along the different push and pull characteristics associated with a criterion, the management of a specific healthcare institution might react differently. Therefore, in the present study, we derive some generic management reactions to handle criteria in each field.

| **Strong pull argument**  (significant odds ratio > 1) | **Paradox**  Trigger criterion, high sample heterogeneity or information asymmetry | **Unfounded attraction**  Unjustified competitive advantage in favour of the organisation | **Prevalence**  Type-specific strength and highly credible competitive advantage |
| --- | --- | --- | --- |
| **Average pull argument**  (non-significant odds ratio) | **Threat**  Underestimated type-specific weakness, expectations not met | **Average**  No competitive advantage or disadvantage, meets industry’s standards. | **Chance**  Underestimated type-specific strength, unabsorbed branding potential |
| **Weak pull argument**  (significant odds ratio < 1) | **Inability**  Type-specific weakness, competitive disadvantage | **Unfounded repulsion**  Unjustified competitive disadvantage at the organisation’s charge | **Irrelevance**  Relatively irrelevant criteria, special assessment needed |
|  | Strong push argument  (significant odds ratio > 1) | Average push argument  (non-significant odds ratio) | Weak push argument  (significant odds ratio < 1) |

***Figure: Work factors as push and pull arguments: Nine strategic reactions***

From the human resource management perspective, a criterion being a below-average push argument while being an over-average pull argument is the optimal case, for example, when stress is a less frequent reason for nurses to leave an organisation but it is a more frequent reason for nurses to join the organisation. We call this field of arguments as *Prevalence* because an organisation benefits from this argument in every respect. It is a type-specific strength and a well-known competitive advantage among nurses, which can be attractive as well as satisfying. The fact that competitors are likely to perform lower regarding this aspect renders it a unique selling proposition for employer branding, which should be emphasised in external and internal communication. Since it is a weak push factor, the pushing of this argument is highly credible and, hence, can be exploited and developed efficiently.

By contrast, *Chance* criteria are significantly less frequent push arguments, yet these are no strong pull arguments. This implies that these factors are undiscovered or underestimated strengths of the organisation, and thus, constitute unabsorbed marketing potential. These factors should be elevated to the *Prevalence* field.

Weak pull arguments while also being weak push arguments fall in the field *Irrelevance* and should be considered as less fortunate. Thus, the criterion is for both, nurses leaving and entering the organisation, relatively irrelevant. This is a result of either a deviant pull or a deviant push characteristic, which should be closely evaluated by observing the corresponding workplace characteristics:

If it is a weak pull argument because—compared to other organisations—the criterion is poorly developed, it may still be a weak push argument because the organisation attracts personalities which are less affected by this criterion. It neither pulls them to nor pushes them away from the organisation. On the one hand, the organisation is lucky to consider the criterion as a flaw without severe consequences, and it should work on the important push factors. On the other hand, it possibly hinders attracting those employees who care about this criterion.

If it is a weak push argument because—compared to other organisations—the criterion is well developed, there are several explanations for the criterion to still be a weak pull argument. First, it might be that a larger number of other nurses who are attracted by stronger pull factors outweighs the number of nurses attracted by the criterion. In this case, the organisation should minimise investments in this criterion since it is not attracting adequate workforce. Second, it might be that nurses do not prefer this organisation although it performs better with regard to the criterion that led to quitting the former organisation. They might avoid this type of organisation due to other important factors that contend against working with this type of organisation. In this case, the organisation should first identify and manage these specific impediments. Third, non-transparency regarding organisational advantages or, possibly, an unjustified reputation spillover problem may be another reason. The specific type of organisation is perceived as bad in some aspects in which it actually performs well. This would mean that communication efforts should be enforced to be, at a minimum, perceived at the same level as other types of organisations. Since, for competitors, it is a rather important pull factor, however, it would be difficult to turn it into a star argument of the in favour of *Prevalence* field. Fourth, it is plausible that some factors, which could be described as dissatisfiers when inexistent or poorly developed would somehow lead to perceived disadvantages, however do not lead to perceived advantages when well-marked. For example, physical or psychological aggression from patients can be a reasonable strong trigger for turnover, while a non-aggressive environment will not be similarly weighted in the assessment of pull arguments, because it is seen as absence of malus instead of a bonus.

*Unfounded attraction* comprises criteria with strong pull and average push characteristics. Thus, nurses who left the former employer type because of a specific criterion consider the same criterion to be satisfying with the organisation portrayed in the diagram (as follow-up employer) although the criterion is as frequently indicated as reason to quit as with other employer types. This may occur due to non-transparency of advantages and disadvantages of the workplace, whereby the organisation seems overrated in terms of this criterion. In this case, the organisation should try to meet the expectations and enhance the criteria in reality in order to use them as credible arguments in employer branding (see *Prevalence*).

Criteria in the *Average* field are characterised by average pull and push performance. This implies that these factors are neither organisational strengths nor weaknesses and should be considered as average important factors that do not call for immediate response and investment by human resource management.

With weak pull and average push characteristics, criteria are allocated to the *Unfounded repulsion* field. Although the criteria only moderately lead to turnover, people who had left a former organisation because of these criteria appear to rather avoid this type of employer. However, it is also possible that the organisation attracts more employees through other, stronger factors. In both the cases, the unpopularity of the criteria as pull arguments seems rather unfounded and should be—although the organisation may never excel in it—promoted to elevate them up to an acceptable reputation that matches actual workplace conditions.

When a criterion with a certain type of employer has strong pull characteristics while also strongly being responsible for turnover, this appears to be a *Paradox*. Yet, this could occur with employees who are strongly attracted by a highly developed criterion but are highly sensitive when their high standards are not met, leading to turnover. Moreover, it could occur in the case of a highly heterogeneous pool of organisations (high variation in the criteria) or nurses (high variation in needs) within one type of employer. Finally, there could be a strong, prior-to-employment overestimation of the quality of these factors with regard of the actual development of the criteria in the organisation. In either case, these factors should receive greater attention since they are strong dissatisfiers and increase costs although they are (potential) genuine pull factors.

Only of an average pull character, yet of a strong push character are criteria in the *Threat* field. These factors are more expensive than are useful in the organisations’ resource management. Although they are strong push factors, they still seem not to burden recruitment since employees who formerly quit a job because of these arguments still are not too discouraged to choose this employer. Since these factors are undiscovered or underestimated weaknesses of the organisation, they must be handled as threats, preventing them by elevating the quality of the criteria to the expected level.

Finally, criteria allocated to the *Inability* field are genuine weaknesses of the organisation. They are significantly more likely to be responsible for turnovers than at other types of organisations, and nurses who are sensitive to these criteria less consider this organisation as a future employer. Since it appears that these factors are connected to innate organisational characteristics, it may be unproductive to go against these weaknesses while it may be more worthwhile to keep them at a tolerable level while investing in factors of other fields.
