# Supplementary material for: Competitive employer positioning through career path analysis: the case of the Swiss nursing sector
Source: Hum Resour Health. 2021 Apr 6;19:47. doi: 10.1186/s12960-021-00586-z (PMC8025559; doi:10.1186/s12960-021-00586-z)
Supplement: Supplementary file 3 — Additional file 3: Strengths-and-weaknesses diagrams for private hospitals, private medical offices, SOMEDs, NPOs, and home care services [file 12960_2021_586_MOESM3_ESM.docx]

## Additional File 3: Strengths-and-weaknesses diagrams for private hospitals, private medical offices, SOMEDs, NPOs, and home care services

##
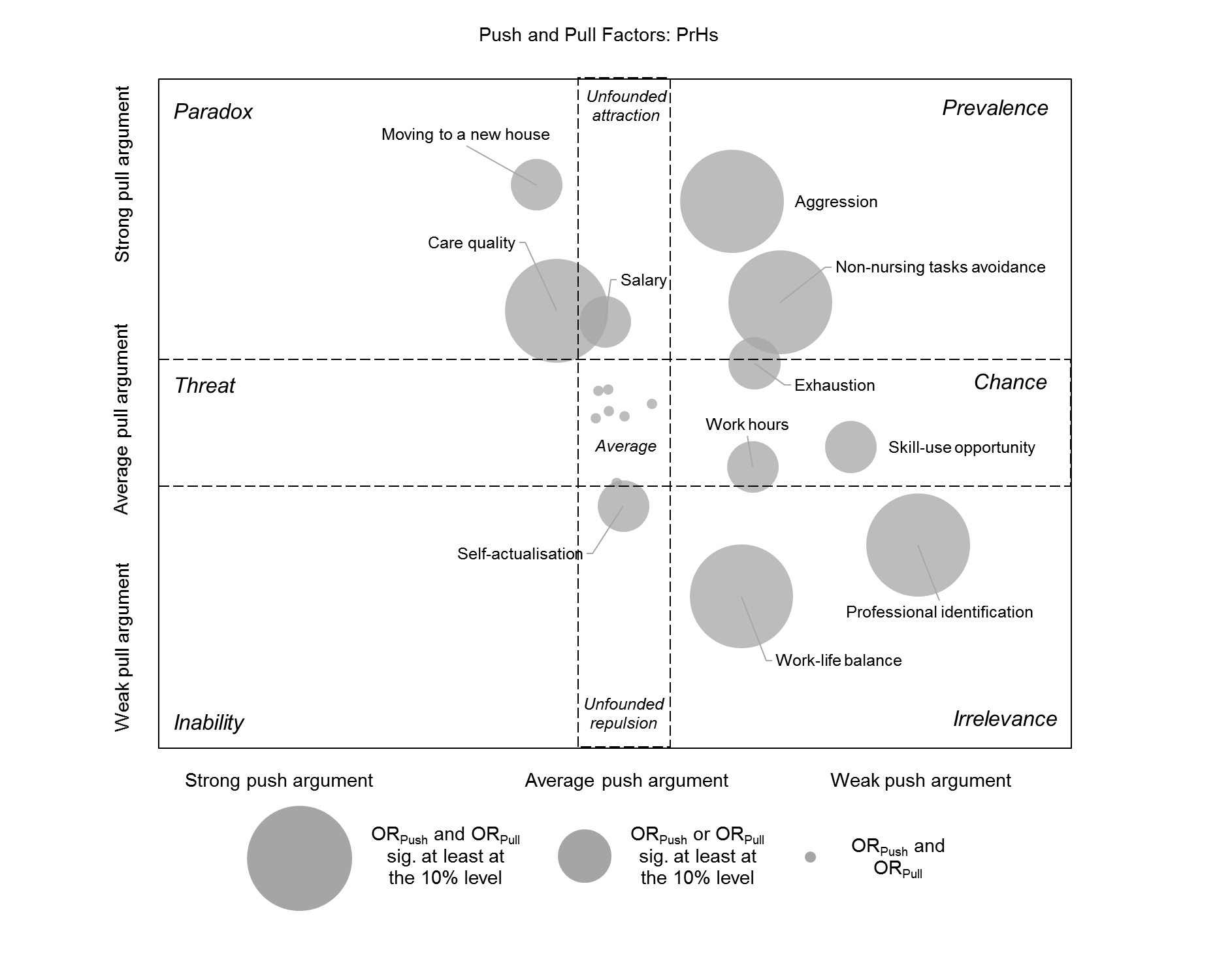


## Figure: Push and pull factors associated with working at private hospitals

Notes: Coordinates based on transformed odds ratios of cluster-robust weighted bivariate logistic regressions determining the push and pull characteristics of each criterion.


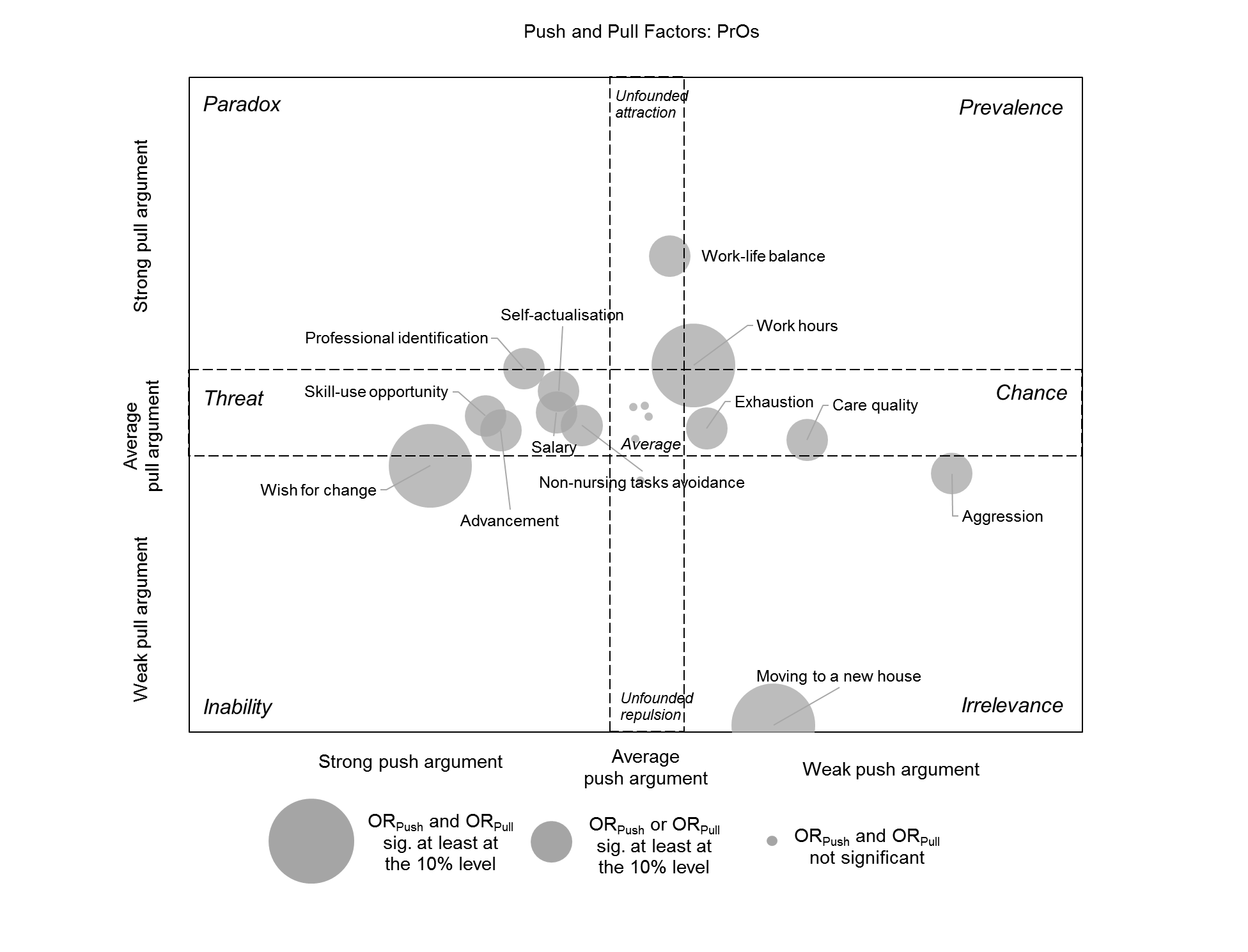


## Figure: Push and pull factors associated with working at private medical offices

Notes: Coordinates based on transformed odds ratios of cluster-robust weighted bivariate logistic regressions determining the push and pull characteristics of each criterion.

##
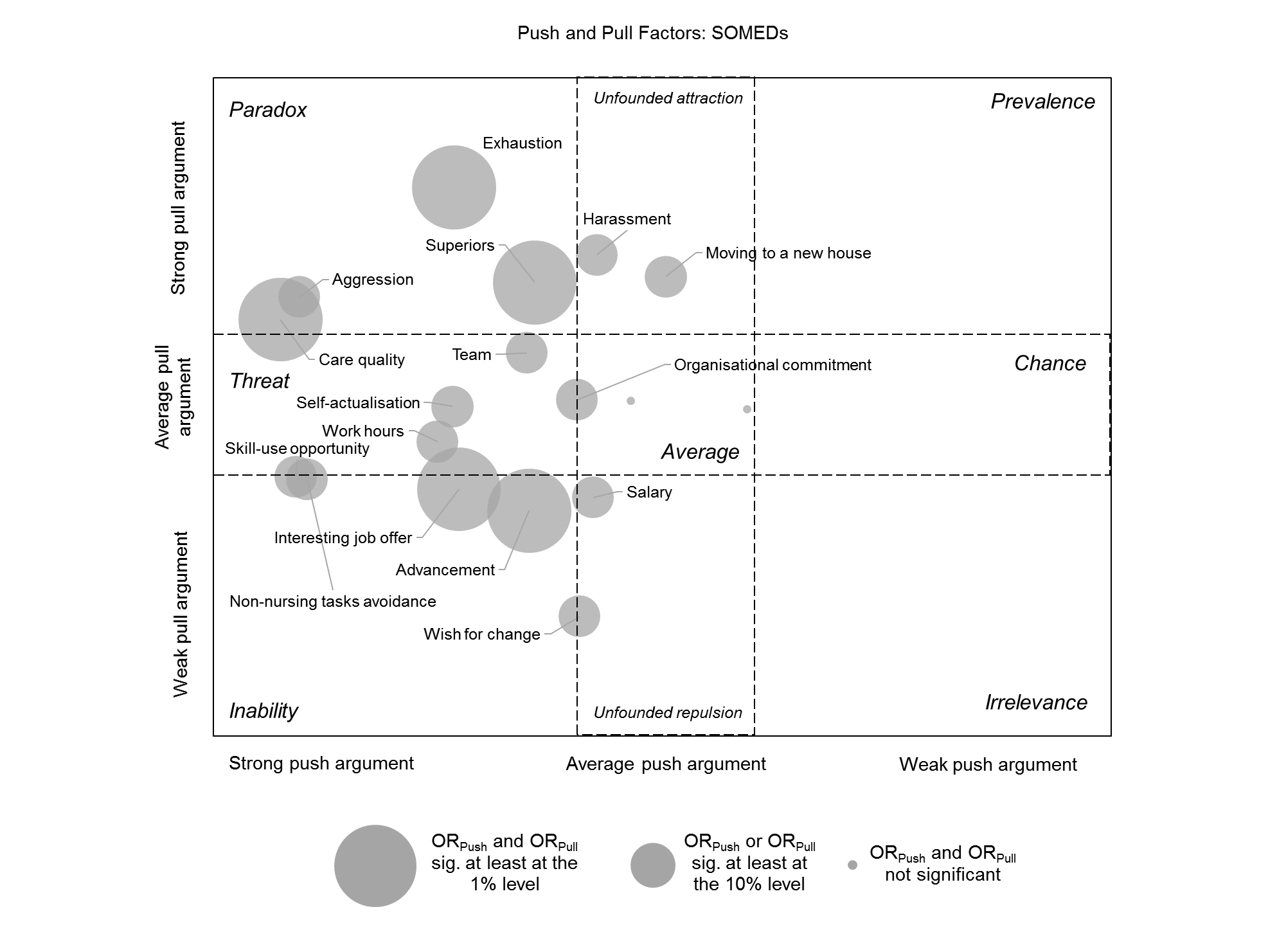


## Figure: Push and pull factors associated with working at socio-medical institutions

Notes: Coordinates based on transformed odds ratios of cluster-robust weighted bivariate logistic regressions determining the push and pull characteristics of each criterion.

##
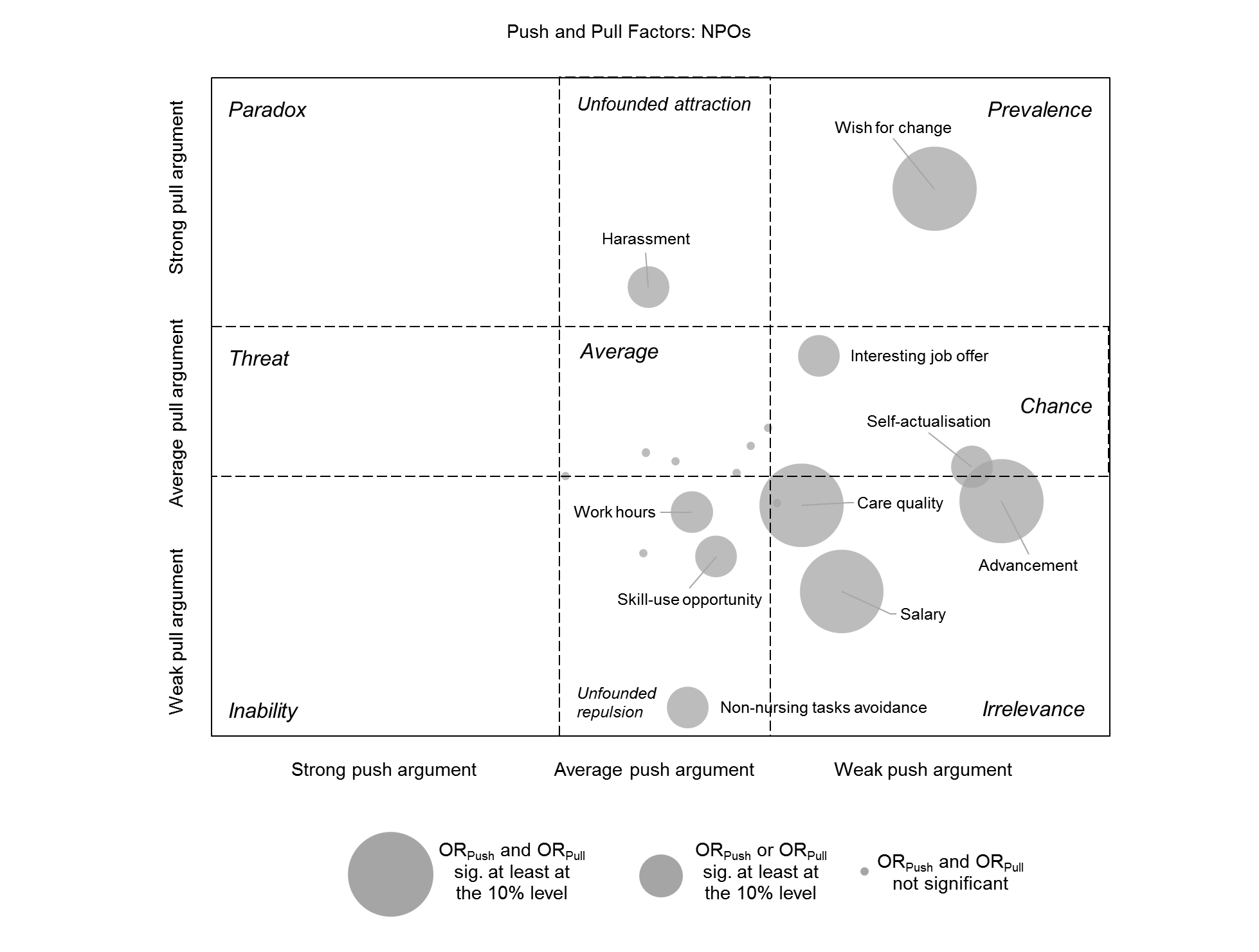


## Figure: Push and pull factors associated with working at non-profit institutions

Notes: Coordinates based on transformed odds ratios of cluster-robust weighted bivariate logistic regressions determining the push and pull characteristics of each criterion.

##
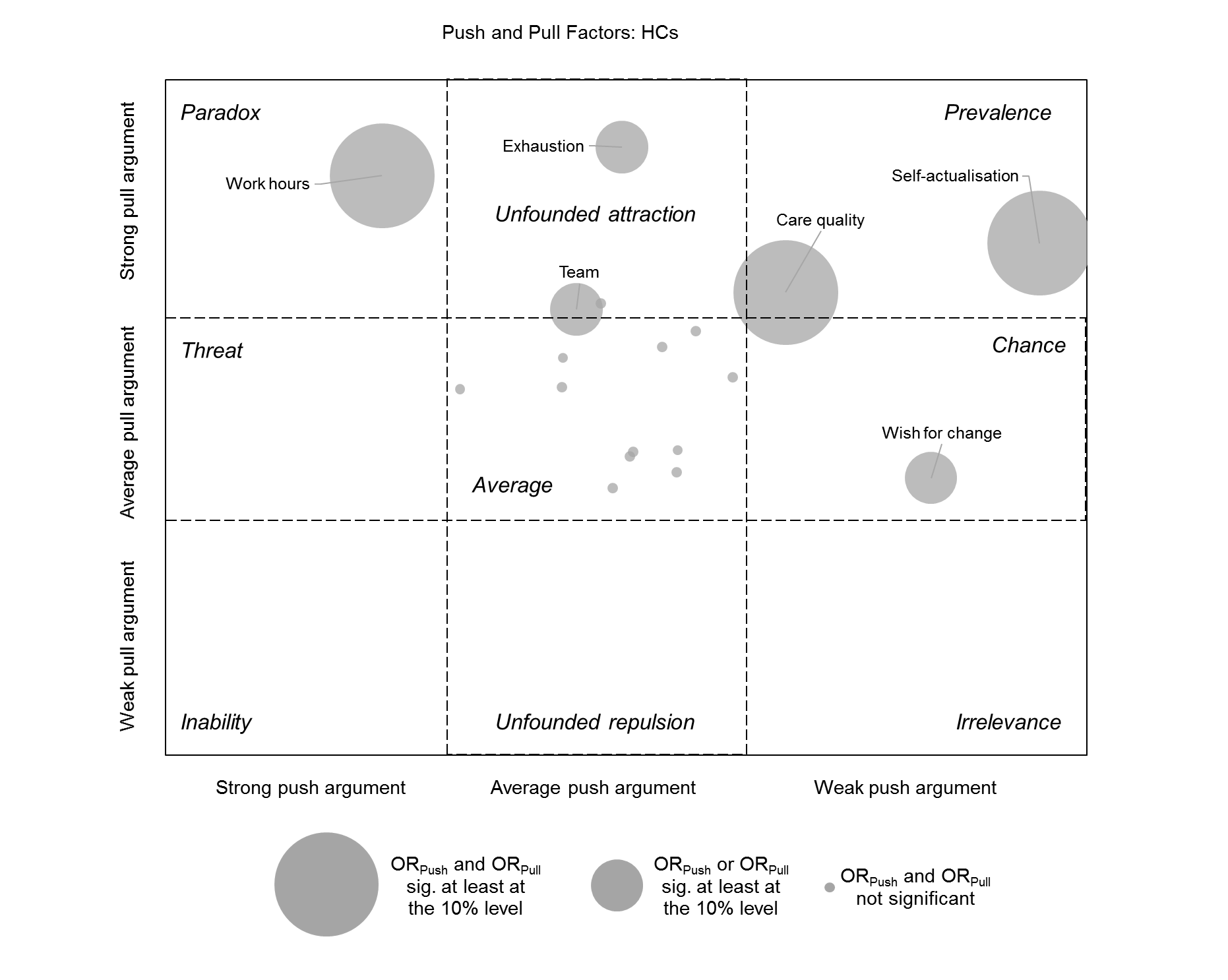


## Figure: Push and pull factors associated with working at home-care institutions

Notes: Coordinates based on transformed odds ratios of cluster-robust weighted bivariate logistic regressions determining the push and pull characteristics of each criterion.
