# Supplementary material for: Competitive employer positioning through career path analysis: the case of the Swiss nursing sector
Source: Hum Resour Health. 2021 Apr 6;19:47. doi: 10.1186/s12960-021-00586-z (PMC8025559; doi:10.1186/s12960-021-00586-z)
Supplement: Supplementary file 4 — Additional file 4: Relevance of reasons to quit involved in cross-typical turnover [file 12960_2021_586_MOESM4_ESM.docx]

***Additional File 4: Relevance of reasons to quit involved in cross-typical turnover***

|  | All turnover cases  (n = 14,244) | | Subsamples of left types of employers:*  Percentage of cases where the reason was indicated | | | | | | |
| --- | --- | --- | --- | --- | --- | --- | --- | --- | --- |
|  | Number of indications | Percentage of cases where the reason was indicated | All  cross-sectoral turnovers  (n = 4,844) | Public hospitals  (n = 2,302) | Private hospitals  (n = 880) | Private medical offices  (n = 142) | SOMEDs  (n = 815) | NPOs (n= 198) | Home care services (n = 507) |
| Professional development wish (RQ7) | 6,439 | 78% | 74% | 71% | 79% | 81% | 79% | 66% | 72% |
| Interesting job offer (RQ12) | 4,358 | 55% | 52% | 51% | 50% | 47% | 59% | 36% | 50% |
| Superiors' support (RQ17) | 3,252 | 41% | 44% | 43% | 44% | 34% | 48% | 39% | 47% |
| Career opportunities (RQ14) | 2,874 | 38% | 39% | 32% | 43% | 63% | 49% | 25% | 40% |
| Participation (RQ10) | 2,871 | 37% | 40% | 38% | 42% | 51% | 46% | 24% | 31% |
| Stress (RQ4) | 2,761 | 35% | 38% | 41% | 30% | 23% | 43% | 29% | 34% |
| Recognition (RQ18) | 2,564 | 32% | 35% | 33% | 36% | 36% | 40% | 25% | 35% |
| Interest in other profession (RQ6) | 2,403 | 32% | 29% | 28% | 26% | 58% | 32% | 26% | 28% |
| Education wish (RQ27) | 2,424 | 31% | 26% | 22% | 33% | 44% | 30% | 15% | 23% |
| Skill-use opportunity (RQ8) | 2,361 | 30% | 33% | 21% | 30% | 67% | 56% | 36% | 38% |
| Work hours (RQ2) | 2,277 | 30% | 34% | 34% | 25% | 21% | 41% | 29% | 40% |
| Training possibilities (RQ13) | 2,226 | 29% | 32% | 24% | 39% | 53% | 42% | 21% | 36% |
| Care quality (RQ3) | 2,192 | 29% | 31% | 32% | 30% | 10% | 39% | 18% | 22% |
| Team mood (RQ15) | 2,105 | 27% | 30% | 28% | 31% | 27% | 35% | 21% | 32% |
| Salary (RQ26) | 2,054 | 26% | 29% | 22% | 34% | 49% | 36% | 21% | 31% |
| Organisational commitment (RQ19) | 1,879 | 24% | 27% | 24% | 29% | 29% | 32% | 22% | 31% |
| Autonomy (RQ9) | 1,865 | 24% | 24% | 22% | 23% | 39% | 32% | 15% | 16% |
| Team cooperation (RQ16) | 1,598 | 20% | 23% | 22% | 21% | 28% | 27% | 19% | 23% |
| Professional exhaustion (RQ24) | 1,433 | 19% | 21% | 25% | 16% | 14% | 21% | 26% | 19% |
| Work-life balance (RQ21) | 1,274 | 17% | 19% | 22% | 13% | 12% | 15% | 25% | 19% |
| Non-nursing tasks avoidance (RQ1) | 1,015 | 15% | 16% | 10% | 14% | 30% | 30% | 18% | 19% |
| Health problems (RQ25) | 848 | 11% | 12% | 14% | 9% | 11% | 12% | 13% | 13% |
| Taking care of children (RQ22) | 597 | 11% | 13% | 14% | 9% | 18% | 9% | 16% | 18% |
| Moving to a new house (RQ23) | 738 | 10% | 10% | 11% | 10% | 3% | 8% | 7% | 8% |
| Mobbing (RQ11) | 797 | 10% | 11% | 11% | 10% | 11% | 13% | 12% | 11% |
| Violence (RQ5) | 606 | 8% | 9% | 10% | 6% | 2% | 13% | 8% | 7% |
| Professional identification (RQ20) | 525 | 7% | 8% | 7% | 5% | 20% | 11% | 7% | 10% |

* Only including turnover cases, where nurses worked at least on full day per week and stayed at least one month for the same employer before they changed the employer. The analyzed subsamples exclude cases where the follow-up employer was of the same type or of a type not listed.
